# The laboratory billing process and its applications to molecular microbiology testing: guidance for laboratorians

**DOI:** 10.1128/jcm.00666-25

**Published:** 2025-09-02

**Authors:** Meghan W. Starolis, Hema Kapoor, Kristen L. Jurcic Smith, Rachael M. Liesman, Dawn R. Zenefski

**Affiliations:** 1 Quest Diagnostics 7172 , Secaucus, New Jersey, USA; 2 HK Healthcare Consultant LLC, Davie, Florida, USA; 3 bioMérieux, Salt Lake City, Utah, USA; 4 Medical College of Wisconsin 5506, Milwaukee, Wisconsin, USA; Vanderbilt University Medical Center, Nashville, Tennessee, USA

**Keywords:** next-generation sequencing, antimicrobial resistance, laboratory billing, reimbursement, syndromic panels

## Abstract

The processes used by laboratories to seek reimbursement for services performed are complex and often unclear to laboratorians, a key group that has the clinical and technical knowledge to influence reimbursement. In this review, we provide a comprehensive overview of laboratory billing processes for insurance billing and hospital inpatient services, as well as the various procedure codes used in these processes. Understanding the health plan landscape is also of critical importance, as health plan policies and coverage decisions have downstream effects on accessibility of testing for patients. A detailed overview of health plans and how coverage determinations are made and communicated is discussed in this review. Lastly, we present actionable areas of opportunity in clinical microbiology where laboratorians, as well as test manufacturers, can focus on improving reimbursement (syndromic panels, antimicrobial resistance, and next-generation sequencing) to expand access to these evolving technologies and encourage adoption in clinical laboratories.

## INTRODUCTION

The availability of diagnostic testing and laboratory services is increasingly influenced by the health plan and billing process landscape. The laboratory billing and reimbursement processes are often opaque and may be poorly understood by laboratorians. Despite routinely making diagnostic testing decisions with laboratory billing implications, most laboratorians learn about the billing and reimbursement processes through on-the-job experiences and may have a limited understanding of the complexity. The purpose of this review is to facilitate laboratory billing and reimbursement navigation by outlining the healthcare plan hierarchy, describing the development and utility of commonly used procedure codes, and identifying persistent challenges that face laboratorians related to test reimbursement, focusing on the molecular microbiology space.

## THE LABORATORY BILLING PROCESS

**Fig 1:**
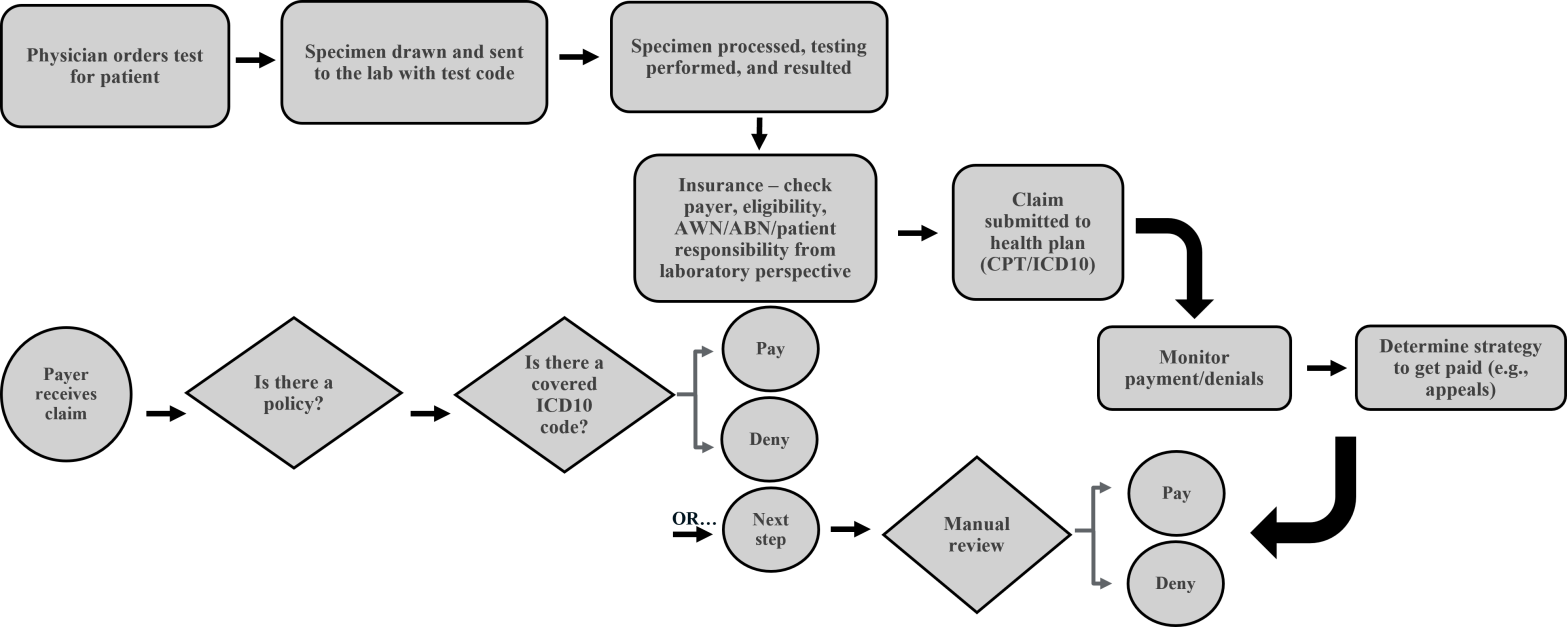
The laboratory billing process.

The laboratory billing process is how a clinical or pathology laboratory seeks reimbursement from a payer (e.g., private or government health insurers) for testing performed (Fig. 1). The process starts when a healthcare provider orders a laboratory test with a corresponding test order code. The healthcare provider submits the diagnosis code, or ICD-10 code (“International Classification of Diseases, Tenth Revision”). ICD-10 codes are an international system published by the World Health Organization for coding diseases and medical conditions. The laboratory receives the order and ICD-10 code(s) and performs the laboratory test(s) as requested. In some cases (e.g., costly molecular oncology or genetic assays), pre-authorization from the health insurer may be required prior to testing, which may add administrative burden to the laboratory to obtain the pre-authorization if not provided. The laboratory assigns each test a Current Procedural Terminology (CPT) or other procedure code that most closely aligns with the test performed. CPT codes are a standardized system of codes developed by the American Medical Association (AMA) and used to describe medical and diagnostic procedures for reporting, billing, and data analysis (1). There are three categories of CPT codes (I–III), which are described in Table 1.

**TABLE 1:** Types of procedure or laboratory codes

Type of code	Description
Category I CPT code	General services (e.g., pathology and laboratory, diagnostic radiology, anesthesia, medicine, evaluation and management, surgery, ophthalmology, and special service codes).
Category II CPT code	Supplemental tracking codes that are not used for billing. These are used for data collection for quality improvement purposes.
Category III CPT code	Emerging technology. These codes are used to track new technologies or services and may eventually be added to category I.
Proprietary Laboratory Analysis code	Codes requested by clinical laboratories or assay manufacturers and assigned by AMA to specifically identify their test when a suitable CPT is not available.
U-code (Healthcare Common Procedure Coding System [HCPCS])	Codes used when laboratory services may not be accurately covered under currently available CPT codes and can be requested by various stakeholders. U-codes are used in the HCPCS.
Logical Observation Identifiers Names and Codes (LOINCs)	LOINCs are used to identify observations in contrast to CPT codes that define procedures. LOINCs can link clinical and billing data and can be used to create CPT codes and other reimbursement codes.
Z-codes	Codes used in conjunction with CPT codes that add specificity to exactly which molecular diagnostic test was performed. The need for Z-codes is determined through MolDX local coverage determinations.

Once testing is complete, the laboratory files a claim with the patient’s health insurer, providing both the CPT code(s) and ICD-10 codes on the claim. Prior to submission, the laboratory may check for eligibility or any Advance Written Notice or Advance Beneficiary Notice of Non-Coverage forms, which are provided to Medicare beneficiaries to disclose that a specific service may not be covered, and the patient may be responsible for payment. Once received by the health insurer, the claim is adjudicated to determine if the CPT code and corresponding ICD-10 code(s) are covered by medical policy. For many payers, this level of review is automated, since these rules are determined by internal coverage policies. The claim will either be paid, denied, or sent for manual review. If the claim is denied, the laboratory can appeal the decision. For example, if no ICD-10 was provided, the laboratory can request one from the ordering physician and re-file the claim. Multiple levels of automated and manual review are possible, which can be time-consuming and costly for both the health insurer and laboratory. If the claim is ultimately denied, the laboratory might bill the patient or absorb the cost of testing, depending on the contracts or policies within the organization.

### Hospital inpatient services

Billing for medical procedures for patients who are admitted to the hospital is through diagnosis-related group (DRG) billing. The DRG billing system was first introduced in 1982 and was developed by Robert B. Fetter (Yale School of Management) and John D. Thompson (Yale School of Public Health) (2). DRG billing is utilized by Medicare as well as some private health insurers. The patient is assigned a DRG based on their ICD-10 diagnoses, with other factors such as age, gender, comorbidities, and required treatment considered (3). The insurer will reimburse the hospital for a set amount based on the average resources typically required for that DRG, regardless of the actual cost of medical services (3). DRGs are further divided into Major Diagnostic Categories, which are 25 different groups comprising all possible principal diagnoses (4).

Diagnostic stewardship is pertinent whether insurance or DRG billing is being utilized. When billing insurance, utilizing medically appropriate laboratory testing increases the likelihood of reimbursement. When using DRG billing, the hospital receives a set reimbursement amount regardless of tests performed. If the cost of procedures exceeds the reimbursement amount, the balance will be covered by the hospital or the patient.

### How CPT codes are created

There may be billing gaps when new technology is developed if no currently available CPT codes accurately describe the assay. Test manufacturers, laboratories, and professional medical societies may need to apply for new CPT codes from the AMA. A category I CPT code, which is the most important for billing purposes, has specific requirements that must be met before an application can be considered. The service must be of sufficiently high volume, although the specific volume is not stated on the Centers for Medicare and Medicaid Services (CMS) website. The service must also be consistent with current medical practice and have clinical efficacy that is published in the literature, which meets requirements in the CPT application (5).

The process for creating a new CPT code can be lengthy (18–24 months) and requires multiple steps (1). First, a medical society, laboratory, or interested group (e.g., American Clinical Laboratory Association) must submit a detailed application to the AMA for the CPT code. The AMA will then determine if the request is new and, if so, refer the application to the CPT advisory committee for evaluation. This process typically takes about 3 months as the AMA must prepare submitted materials for the Editorial Panel, and CPT advisors must respond and ensure all required information from the applicant has been provided. The CPT Editorial Panel meets three times per year and reviews an agenda with applications and CPT advisor comments. The Editorial Panel votes to approve or deny the request and determines the appropriate category if applying for a category I or III code. The Editorial Panel may also refer the request to a workgroup for further study or postpone the vote to a future meeting. The AMA staff will inform the submitter of the panel decision. If the CPT code is approved as category I, it becomes effective on 1 January of each year. Category II codes are released three times per year at set dates and are effective 3 months after the release date. Category III codes are released 1 January or 1 July and become effective 6 months after the release date.

### Other types of procedure codes

There are other types of procedure codes that may be used in the billing process (Table 1). The first is called a Proprietary Laboratory Analysis (PLA) code, an alphanumeric code requested by clinical laboratories or assay manufacturers to identify their specific test (6). If a laboratory or manufacturer is granted a PLA code, any laboratory running this assay will use the PLA code to bill (7). Applications for PLA codes are submitted to AMA and are discussed by the CPT Editorial Panel. One major difference in PLA vs CPT codes is that PLA code applications do not require evidence of clinical utility. Once sufficient clinical utility data have been collected and there is significant testing volume, a category I CPT application can be considered. While having a PLA code may help with reimbursement when there is no suitable CPT, they are not covered as widely as category I CPT codes, given that they are special billing codes created or used by specific laboratories as opposed to standardized CPT codes. Another type of reimbursement code is called a U-code under the Healthcare Common Procedure Coding System (HCPCS) (8). These codes are used when laboratory services may not be accurately covered under currently available CPT codes. U-codes can be created more rapidly as they are updated throughout the year based on feedback from various stakeholders. For example, early in the COVID-19 pandemic (2020), U-codes were released for reimbursement of SARS-CoV-2 PCR testing prior to the creation of category I CPT codes (code U0001 for Centers for Disease Control and Prevention [CDC] testing followed by U0003 for non-CDC testing). However, there is potential for denials when using U-codes. U-codes are accepted by Medicare, Medicaid, and some private insurers but are not universally accepted by all payers, increasing the risk of denials for U-codes compared to CPT codes (9).

Another type of reimbursement code specific to molecular diagnostic tests is Z-codes, which are unique five-character alphanumeric codes used in the laboratory billing process to identify the specific test that was performed. Z-codes are assigned by the Diagnostics Exchange (DEX), a molecular diagnostic test identification and policy management system used by Palmetto GBA, a company that provides administrative services to the federal government. Z-codes were first used in 2018 for certain molecular genetics and oncology assays. A unique feature of Z-codes is that they are specific to the laboratory or manufacturer who requested the code, although another organization can request sharing of the Z-code once granted. MolDX, a program that reviews and registers molecular diagnostic tests for Medicare, determines coverage and the reimbursement amount for a specific Z-code. In 2023, MolDX released the Local Coverage Determination (LCD) L38988, titled “MolDX: Molecular Syndromic Panels for Infectious Disease Pathogen Identification Testing,” which specified combinations of CPT codes to define a syndromic panel and required Z-codes for Medicare reimbursement in MolDX jurisdictions (discussed in more detail below in the section “Syndromic Panels”). MolDX policies change frequently, leading to ongoing challenges with interpretation among healthcare providers, billing departments, and laboratorians.

MolDX has seven jurisdictions (JM A/B, JJ A/B, JE A/B, JF A/B, J5 A/B, J8 A/B, and J15 A/B) for payers in 30 U.S. states and territories (a full list of affected areas and administrators can be found on the Palmetto GBA MolDX Program website Frequently Asked Questions [FAQs]) (10). It is important to note that the geographic location of the performing laboratory determines the MolDX jurisdiction. All private, reference, and hospital laboratories that perform molecular diagnostic testing for syndromic panels and submit claims to Medicare in JE, JF, JJ, JM, J15, J5, or J8 on forms CMS 1500 (part B), UB04 (part A), or electronic claims on a 5010-837P (part B) or 837I (part A) must submit for and bill with appropriate Z-codes. It is also notable that private payers are starting to require Z-codes. UnitedHealthcare began requiring Z-codes for many molecular diagnostic tests as of August 2024, and Humana began requiring them in September 2024. Other major insurers are expected to follow suit. This will expand the number of laboratories that must utilize Z-codes in the billing process.

To obtain a Z-code, a laboratory or manufacturer must first register with DEX (https://app.dexzcodes.com). Once registered, the laboratory must complete an application for a Z-code, which includes, but is not limited to, the test code and name, description of the testing, performing laboratories, molecular analyte detected, Food and Drug Administration (FDA) document number (if applicable), methodology, and CPT code(s). Once submitted, DEX will review the application and assign a Z-code. A separate submission to MolDX is required to establish the coverage and reimbursement amount for the Z-code. Test requisitions, standard operating procedures, and sample reports are included in the application to MolDX. A technical assessment with data to support clinical and analytical validation data and clinical utility is required for laboratory-developed tests or modified FDA-approved tests. If a laboratory sends testing out to a reference laboratory, both laboratories must be registered with DEX, but only the performing site needs to obtain the Z-code. When submitting a reimbursement claim, the laboratory submits the CPT code, ICD-10 code(s) provided by the ordering physician, and the Z-code.

### Considerations for medical coding

Deciding what reimbursement codes should be used for any given test should be done by a person experienced in medical coding in coordination with individuals knowledgeable about the testing methodology to avoid costly billing errors. Health plans often follow the National Correct Coding Initiative (NCCI), a program developed by CMS to promote correct coding. Incorrect coding may trigger NCCI Procedure to Procedure (PTP) edits, a type of edit that prevents inappropriate payment of services that should not be reported together. Another type of NCCI edit is Medically Unlikely Edit (MUE), which is used by Medicare Administrative Contractors (MACs) to define the maximum units of service for a specific CPT or HCPCS code that can be billed (11).

An example of NCCI PTP and MUE edits is highlighted in the following example for respiratory virus testing. If a laboratory created a reflex from a multiplex influenza A/B/RSV test (bills with CPT 87631) to an influenza A subtyping test and attempted to bill with CPT 87503 (a CPT code used for additional Influenza subtypes after the first two subtypes are detected), an NCCI PTP edit would trigger, resulting in denial of CPT 87503 (Table 2). CPT 87503 must be billed in conjunction with CPT 87502 and has an MUE of 1, meaning that only one unit of 87503 can be billed. If more than one subtype was tested and billed with 87503, only one instance of 87503 would potentially be reimbursed, or the entire claim could be denied. In this example, although coding was correct, billing restrictions on code combinations or CPT usage limits could still lead to denied claims. The laboratory could choose to accept the potential for denied claims or restructure the proposed testing algorithm to something more favorable for reimbursement.

**TABLE 2:** Examples of CPT codes for influenza testing

CPT codes	Description
87502	Test to determine the presence of multiple types or subtypes of influenza virus through nucleic acid detection using the multiplex amplified probe technique and possibly multiplex reverse transcription. Use this code to report the first two types or subtypes.
87503 (must be used in conjunction with 87502)	Test to determine the presence of the influenza virus through nucleic acid detection, including multiplex amplified probe technique and possibly multiplex reverse transcription for each additional influenza virus type or subtype after the detection of the first two types or subtypes.
87636	Infectious agent detection by nucleic acid (DNA or RNA). SARS-CoV-2 and influenza virus types A and B, multiplex amplified probe technique.
87631	Respiratory virus, 3–5 targets through nucleic acid detection using multiplex amplified probe technique and possibly multiplex reverse transcription.
87501	Influenza virus includes reverse transcription and amplified probe tech, each type or subtype.

While it is the responsibility of the performing laboratory to properly code, coverage decisions are made by payers through payer-specific policies such as LCDs or national coverage determinations (NCDs). LCDs/NCDs are coverage policies that specify which reimbursement codes are covered, along with the specific ICD-10 diagnoses. Even if a laboratory bills with appropriate procedure codes for a test ordered by a licensed healthcare provider, the claim may still be denied based on health plan policies. In some cases, there may be insufficient clinical evidence to support the use of that test for the specific clinical scenario. Strategies for laboratories in dealing with denials are discussed in more detail below.

## OVERVIEW OF HEALTH PLANS

The health plan landscape influences both the availability and utilization of diagnostic services and plays a significant role in shaping the accessibility, cost, and utilization of laboratory testing. A health plan, or payer, is the entity responsible for covering the cost of medical services. How services are utilized and reimbursed depends in part on the payer type (e.g., traditional Medicare/Medicaid, managed care plans, or private insurers) (Table 3). Additional factors include pricing pressures from government-sponsored and commercial plans (new ventures), fewer exclusive contracts for laboratory services with major commercial plans (e.g., Aetna and United Health Care [UHC]), ongoing legislation that reduces CMS reimbursement such as Protecting Access to Medicare Act, and third-party intermediaries (e.g., Avalon and eviCore) who manage medical policy and payer claims adjudication. There is also a shift from “fee-for-service” models, where healthcare providers are incentivized based on volume of services provided, to a “pay-for-performance” model in which payment is tied to quality and efficiency of provided services. Medicare, Medicaid, and private insurance plans each have their own set of rules regarding coverage, reimbursement rates, and required documentation. Understanding the hierarchy and requirements of each health plan is crucial for laboratories to ensure appropriate reimbursement.

**TABLE 3:** Health plan overview

Type of payor	Type of plan		Description
Government (public)	Traditional	Medicare	Federally funded and managed by the CMS (for age 65 and older or under 65 with specific disabilities, e.g., end-stage renal disease).
		Medicaid	Health coverage for low-income individuals is managed by CMS and budgeted by individual states.
		Medicare-Medicaid Dual Eligible	Health coverage for 65 and older for low-income individuals. Medicaid plans will pay first, followed by Medicare.
		CHIP^*b*^	Each state runs its own CHIP program, with broad guidance and funding from the CMS, for eligible low-income uninsured children and women whose income is too high to qualify for Medicaid.
		Tricare	For active-duty military, National Guard/Reserve members or retirees, and their families, it is managed by the Defense Health Agency under the leadership of the Assistant Secretary of Defense (Health Affairs).
	Managed	Medicare Advantage	For age 65 and older.
		Managed Medicaid	Provides Medicaid health benefits through contractual arrangements between state Medicaid agencies and managed care^*a*^ organizations (MCOs) that accept a set per member per month (capitation) payment for these services.
Private/commercial	Health maintenance organization (HMO)		Covers only the cost of medical services involving an in-network doctor or hospital, except for emergency care. To see a specialist, a referral must be obtained from the primary care practitioner (PCP).
	Point of service		Similar to an HMO plan, a referral from the PCP is required to see a specialist. This plan’s higher premiums allow it to cover out-of-network doctors.
	Preferred provider organization		A type of plan in which the subscriber pays less out-of-pocket fee for seeing doctors, hospitals, and other healthcare providers that belong to the plan’s network. It offers more flexibility in accessing healthcare services, including laboratory tests.
	Independent physician association		An IPA is a business entity organized and owned by a network of independent physician practices to reduce overhead or pursue business ventures, such as contracts with employers, accountable care organizations, and/or MCOs.

^*a*^ Managed care is a healthcare delivery system that manages cost, utilization, and quality.

^*b*^ CHIP, Children's Health Insurance Program.

Health insurance, both public and private, covers most of the U.S. population and is the basis for services rendered. Health expenditures rose significantly from $1.4 trillion in 2020 to $4.9 trillion in 2023 (12). In 2023, private insurance expenditures accounted for approximately 30% of total health spending, with public insurance accounting for approximately 43% (13). The hierarchy of health plans in the U.S. is structured to accommodate various types of coverage, each with its own set of rules and benefits. At the top of this hierarchy are traditional Medicare and Medicaid public programs, which are government-funded programs providing coverage primarily for the elderly, disabled, and low-income individuals. CMS relies on a network of multi-state, regional MACs to process medical claims for beneficiaries. In many cases, the test must meet the criteria set forth in the LCDs by local MAC jurisdictions (14) or NCDs set forth by CMS. Criteria used for determining coverage include evidence of clinical utility (i.e., improved patient outcomes) and clinical validity (i.e., the performance of the test itself). Laboratories should ensure that their procedure codes, such as CPT codes, align with these policies to avoid payment denials.

Below traditional Medicare and Medicaid are Medicare Advantage and Managed Medicaid plans, which are administered by private insurers but follow Medicare and Medicaid guidelines. A patient may have a supplemental Medicare Advantage or Managed Medicare plan in addition to Medicare/Medicaid, or these can be purchased as stand-alone policies. These plans may have additional requirements or restrictions set forth by a private payer, making the billing process more complex. While CMS establishes the basic coverage requirements, commercial insurers managing Medicare Advantage plans can add their own limitations (e.g., utilization management review) and policies in addition to existing guidelines. This can result in more restrictive coverage compared to traditional Medicare, affecting the accessibility and affordability of testing.

Private payers, or commercial health plans, including employer-sponsored insurance and individual market plans, form the next layer (Table 3). According to the U.S. Census Bureau, more Americans have a private health plan (e.g., Aetna, Humana, United Healthcare, and Cigna) than public plans (Medicare and Medicaid) (15). These plans are diverse, with each insurer having its own set of coverage policies, deductibles, and payment schedules. To add complexity, many health plans utilize laboratory benefit managers, or intermediaries, to set policy, manage utilization, and/or adjudicate claims. At the base of the hierarchy are specialized health plans, such as short-term health insurance, supplemental insurance, and catastrophic plans.

## MOLECULAR MICROBIOLOGY REIMBURSEMENT CHALLENGES

### Syndromic panels

Advances in multiplexed molecular technologies have led to the development and adoption of syndromic testing approaches, where many organisms that cause similar clinical presentations are detected in one reaction. This has resulted in improvements, such as reduced time to diagnosis, fewer required specimens, and simplified ordering processes (16). Syndromic panels can vary in the choice and number of targets offered by each manufacturer (17). Currently, CPT reimbursement for panels is specific to syndromes (e.g, gastroenteritis, respiratory viral disease, meningitis/encephalitis, and bloodstream infections) and based on how many targets are detected. Panel size options for CPT codes currently available include 3–5 targets, 6–11 targets, and 12–25 targets (18). Coverage policies for syndromic panels are restricted to specific ICD-10 diagnosis codes and, in some cases, must be ordered by a specialist such as an infectious disease physician or gastroenterologist. MolDx MACs and several private payers are now requiring Z-codes for reimbursement of molecular syndromic panels (19).

### Antimicrobial resistance

Molecular detection of antimicrobial resistance (AMR) markers has become commonplace in the clinical laboratory with benefits such as improved turnaround time and sensitivity compared to phenotypic methods, aiding in epidemiological investigations and AMR surveillance (20). Despite these benefits, reimbursement for the detection of AMR targets remains a challenge. There is no organism-agnostic CPT code available for resistance markers, which may deter their implementation in the clinical laboratory and their development by manufacturers. For molecular assays that detect the organism along with markers of resistance, reimbursement may be limited to the detection of the organism, which may not fully cover the cost of testing. Available AMR CPT codes are currently limited to a small number of specific organism/antibiotic combinations. In January 2025, CPT code 87513 for *Helicobacter pylori* detection and clarithromycin resistance and code 87564 for *Mycobacterium tuberculosis* detection and resistance markers to rifampin became available. Another example is CPT 87641 for the detection of methicillin-resistant *Staphylococcus aureus*. Given the lengthy process for creation of CPT codes, having codes that are specific to the organism and the antibiotic is insufficient to encourage assay development in this space.

### Sequencing

Sequencing techniques, especially innovations such as next-generation sequencing (NGS) and metagenomic NGS (mNGS), present opportunities to improve infectious disease diagnosis (21). Targeted sequencing can expand the number of detectable targets when using a syndromic testing approach or when testing markers of resistance, whereas unbiased mNGS allows for broad pathogen detection (21). While there are several technical and interpretive hurdles to overcome, there are also reimbursement challenges that deter innovation in this area (22). The cost for sequencing is high compared to molecular techniques such as PCR due to the cost of reagents, instruments, and bioinformatics resources. A specific CPT code is available for HIV-1 genotyping of reverse transcriptase and protease regions (87901), although it is not specific to sequencing as a methodology. Another example is CPT 87910 for the genotype of *Cytomegalovirus*, which is not specific to sequencing but may be used when performing sequencing-based tests. To date, there is no CPT code for unbiased mNGS (22). There are commercial laboratories offering clinical metagenomics testing for infectious disease diagnosis, but the high cost, lack of reimbursement, and complex interpretation have limited the use of these assays.

### Dealing with denials

There are several ways that laboratorians can contribute to improvements in reimbursement. The first, and arguably most important, step is understanding the denial rates broken down by payer within the institution, partnering with billing or finance departments, and understanding the reasons for denial (e.g., inappropriate coding, NCCI PTP edits, MUE limits, testing in patient populations outside of approved policies). When testing is being ordered for inappropriate patient populations, laboratorians in academic or community health systems can work with the physicians within the health system on diagnostic stewardship for testing without demonstrated clinical utility for a given clinical scenario. Educational efforts (e.g., webinars and presentations at Grand Rounds) can influence test ordering practices, but electronic interventions prior to test ordering, such as Best Practice Alerts, gatekeeping, order sets, and setting up reflex testing and panels in the electronic medical record ordering systems, may be necessary (23). Additionally, professional societies, such as the American Society for Microbiology and the Association for Molecular Pathology, offer opportunities for laboratorians to serve on committees that develop clinical practice guidelines or best practice documents aimed at promoting stewardship. In some cases, such as emerging technologies or new clinical indications, laboratorians can influence reimbursement by generating clinical utility data through controlled studies and collaborating with colleagues in market access and reimbursement to advocate for broader coverage. Individuals with expertise in market access and reimbursement may be employed by the clinical laboratory, the assay manufacturer, or a professional society.

### How laboratories and test manufacturers can collaborate on reimbursement

Partnership between laboratories and manufacturers to generate clinical utility data can lead to many positive outcomes regarding reimbursement. Reimbursement challenges can be minimized if there is collaboration between laboratories and manufacturers of *in vitro* diagnostic assays in the earliest stages of development. An established reimbursement strategy will encourage laboratory adoption of new assays or methods. Laboratories and manufacturers can collaborate on studies to generate the clinical validity data needed to justify additional reimbursement codes for new assays. Collaborative studies can also provide insights on patient outcomes, improved diagnoses, changes in patient management, algorithmic testing methods, appropriate patient populations for testing, and economics associated with improved healthcare outcomes. These data generation can be a shared responsibility between laboratories and manufacturers and, in some cases, may be financially supported by the manufacturers through investigator-initiated studies and other methods of collaboration (24). Manufacturers who have Market Access Teams, which include individuals experienced with reimbursement and medical coding, can help laboratories with appropriate coding and preparation for conversations with payers regarding reimbursement gaps. Some manufacturers also have government affairs teams that help influence the coding environment at the policy level.

## SUMMARY

In laboratory medicine, there is a consistent need for innovation due to emerging and re-emerging pathogens, the need to improve time to diagnosis for timely clinical intervention, and evolving patient needs (e.g., self-collection and home testing). However, the adoption of new technologies such as multiplex molecular testing, NGS, and detection of AMR genes in the clinical laboratory is an ongoing challenge due to reimbursement pressures. When new technologies are released without corresponding reimbursement codes, peer-reviewed literature on clinical performance, or FDA approval, laboratories may be unwilling to adopt testing. Understanding the laboratory billing landscape, actively participating with AMA in the creation of new CPT or PLA codes, working closely with billing departments and/or payers on frequent denials, and collaborating with manufacturers early in the development process are all productive ways of positively changing the reimbursement landscape.
